# Racial differences in postpandemic trends in prostate-specific antigen screening

**DOI:** 10.1093/jncics/pkae016

**Published:** 2024-03-28

**Authors:** Zhiyu Qian, Jameshisa Alexander, Danesha Daniels, Firas Abdollah, Alexander P Cole, Hari S Iyer, Quoc-Dien Trinh

**Affiliations:** Department of Urology, Brigham and Women’s Hospital, Harvard Medical School, Boston, MA, USA; Center for Surgery and Public Health, Brigham and Women’s Hospital, Harvard Medical School, Boston, MA, USA; Department of Urology, Brigham and Women’s Hospital, Harvard Medical School, Boston, MA, USA; Center for Surgery and Public Health, Brigham and Women’s Hospital, Harvard Medical School, Boston, MA, USA; Department of Urology, Brigham and Women’s Hospital, Harvard Medical School, Boston, MA, USA; Center for Surgery and Public Health, Brigham and Women’s Hospital, Harvard Medical School, Boston, MA, USA; Vattikuti Urology Institute Center for Outcomes Research, Analytics and Evaluation, Henry Ford Hospital, Detroit, MI, USA; Vattikuti Urology Institute, Henry Ford Hospital, Detroit, MI, USA; Department of Urology, Brigham and Women’s Hospital, Harvard Medical School, Boston, MA, USA; Center for Surgery and Public Health, Brigham and Women’s Hospital, Harvard Medical School, Boston, MA, USA; Section of Cancer Epidemiology and Health Outcomes, Rutgers Cancer Institute of New Jersey, New Brunswick, NJ, USA; Department of Urology, Brigham and Women’s Hospital, Harvard Medical School, Boston, MA, USA; Center for Surgery and Public Health, Brigham and Women’s Hospital, Harvard Medical School, Boston, MA, USA

## Abstract

Our study investigates the trends in prostate cancer screening amid the COVID-19 pandemic, particularly focusing on racial disparities between Black and White men. Utilizing data from the Behavioral Risk Factor Surveillance System from 2018, 2020, and 2022, we analyzed prostate-specific antigen screening rates in men aged 45-75 years. Our findings reveal initial declines in screening rates for both groups during the pandemic, with subsequent recovery; however, the pace of rebound differed statistically significantly between races. Whereas White men showed a notable increase in screening rates postpandemic, Black men’s rates recovered more slowly. This disparity underscores the impact of socioeconomic factors, health-care access, and possibly systemic biases affecting health-care delivery. Our study highlights the need for targeted interventions to address these inequalities and ensure equitable access to prostate cancer preventive care in the aftermath of COVID-19.

The onset of COVID-19 in March 2020 brought unprecedented disruptions to health care in the United States, profoundly impacting preventive cancer screenings (1-3). These disruptions were not evenly distributed across populations, with evidence pointing to a disproportionate impact on Black communities (4). As the health-care system gradually adapts and evolves in response to the pandemic, there are emerging indications of a resurgence in preventive services (5). This is particularly relevant in the context of prostate cancer, a disease that disproportionately affects Black men because of a combination of biological and socioeconomic factors (6). However, there has been a lack of investigation of the trends in prostate cancer screening in the postpandemic era, especially in relation to racial disparities. We aim to fill this critical gap in literature.

We identified Black and White men aged 45-75 years from the Behavioral Risk Factor Surveillance System database, from the 2018, 2020, and 2022 surveys. This age cohort was selected based on recommendations from the most recent National Comprehensive Cancer Network guidelines for early detection of prostate cancer. The primary outcome was the incidence of prostate-specific antigen (PSA) screening for prostate cancer 2 years prior to each survey period. Respondents from states lacking longitudinal data during the study period were excluded. Baseline demographic characters were calculated. We employed adjusted logistic regression models, incorporating a 2-way interaction term between race and survey year, to explore temporal trends in PSA screening among Black and White men. Statistical significance levels were set at a *P* value less than .05. Analysis was performed using Stata version 17. The study was performed under a general institutional review board protocol for using anonymized publicly available surveys.

Our study included a weighted total of 1.32 million men. Demographic information is detailed in supplementary materials (Supplementary Table 1, available online). In our multivariable analysis, a significant interaction was observed between race and survey year (*P*_interaction_ = .01). Adjusted rates of PSA screening for White men were 45.2%, 41.2%, and 52.7% from 2018, 2020, and 2022, respectively. For Black men, these rates were 55.1%, 43.2%, and 52.5%, respectively (Figure 1). Between 2018 and 2020, PSA screening declined for White men (adjusted odds ratio [AOR] = 0.83, 95% confidence interval [CI] = 0.72 to 0.98) and Black men (AOR = 0.65, 95% CI = 0.48 to 0.88). Conversely, from 2020 to 2022, we saw an increase in screening in White men (AOR = 1.62, 95% CI = 1.39 to 1.92), however, Black men did not exhibit statistically significant changes (AOR = 1.35, 95% CI = 0.96 to 1.89) (Table 1). Analyses of marginal effects revealed statistically significant differences in the magnitude of change between White and Black men in 2022 in reference to 2018 (*P* = .148 for 2020 and *P* = .007 for 2022).

**Figure 1.**
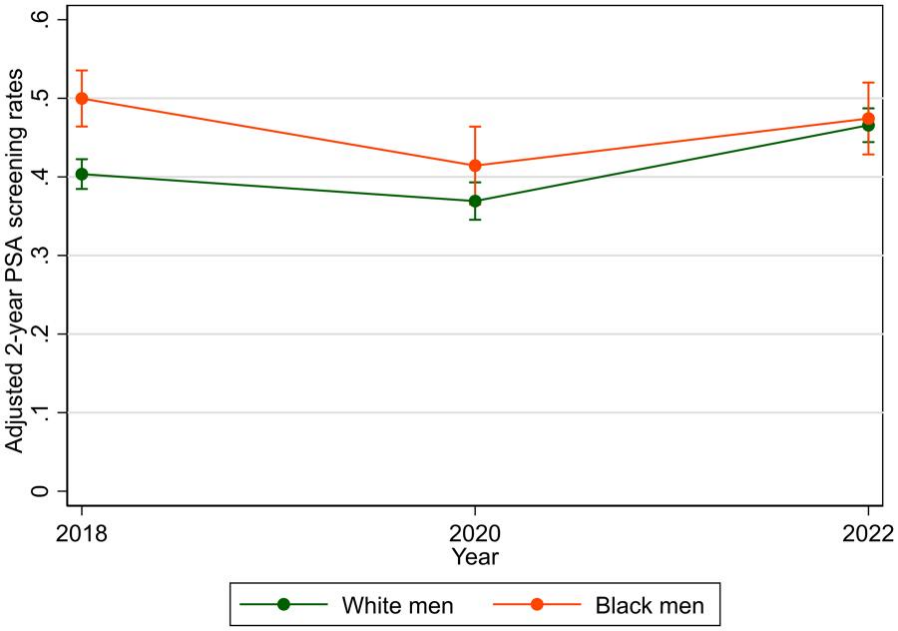
Adjusted 2-year prostate-specific antigen screening rates from 2018 to 2022 among Black and White men aged 45-75 years. Covariates adjusted for in the model included age at the time of survey, education level, annual income, insurance coverage, marital status, smoking status, self-reported overall status of health, and having a personal doctor or not. Only states with longitudinally recorded PSA from 2018-2022 were included. PSA = prostate-specific antigen.

**Table 1.** Adjusted odds ratios for 2-year prostate-specific antigen screening between Black and White men aged 45-75 years from 2018 to 2022

Year	OR for Black men (95% CI)^a^	OR for White men (95% CI)	*P* between Black and White men
2018	Referent	Referent	
2020	0.65 (0.48 to 0.88)	0.83 (0.72 to 0.98)	.148
2022	0.88 (0.66 to 1.17)	1.42 (1.11 to 1.83)	.007

a Covariates adjusted for in the model included age at the time of survey, education level, annual income, insurance coverage, marital status, smoking status, self-reported overall status of health, and having a personal doctor or not. Only States with longitudinally recorded PSA from 2018-2022 were included. CI = confidence interval; OR = odds ratio.

Our analysis of national data reveals a worrisome trend in PSA screening rates during and after the COVID-19 pandemic. Initially, we observed a decrease in screening rates for Black and White men from 2018 to 2020, followed by an increase from 2020 to 2022. However, the rates of change differed between these groups with statistical significance. Although both experienced declines during the early pandemic, Black men demonstrated a notably slower recovery in the postpandemic period. Our findings are consistent with literature suggesting that racial minorities, particularly Black men, were more likely to experience health service disruptions during the initial phase of the COVID-19 pandemic (2,4,7). Our findings showcased that although utilization of preventive care in the United States rebounded following COVID-19 lockdowns, Black men could face greater barriers in recovering prepandemic rates of prostate cancer preventive care.

The disparity in rebounds of PSA screening rates in Black men are likely influenced by a myriad of interrelated factors beyond socioeconomic disadvantages and structural racism. Factors such as a tendency to utilize underresourced hospitals, a history of medical mistrust, and employment in essential jobs with limited health leave could also contribute (4,7-9). As screening guidelines increasingly support PSA testing, it becomes imperative to develop targeted interventions addressing these specific challenges, especially in high-risk populations.

Hence, targeted policies are needed. These could involve increasing funding for community health initiatives specifically designed to reach Black men and improving the accessibility of health-care services in underserved areas. Policies should also focus on rebuilding trust within these communities, emphasizing culturally sensitive care and patient education. The integration of telemedicine and digital health solutions, amplified during the COVID-19 pandemic, presents another potential opportunity to bridge these gaps. Leveraging technology could provide more accessible options, particularly for communities that traditionally face barriers to health-care access.

Our findings are not without limitations. First, our data from a national survey conducted mainly via phone was subject to inherent recall and sampling bias. However, the strength of our study stemmed from our use of the largest continuous health survey maintained by the Centers for Disease Control and Prevention, leading to a diverse population with robust statistical power. Although there are some small differences in screening rates compared with other national surveys like the National Health Interview Survey, our findings align with historical BRFSS data, emphasizing the reliability of our analysis (10). This underscores the value of considering different survey methodologies and demographic samples in health research. Lastly, we acknowledge that self-reported data may not perfectly mirror broader population trends, which is an inherent limitation of survey-based research.

In conclusion, our study highlights a critical disparity in post-COVID-19 PSA screening recovery between Black and White men. Black men’s recovery lags, underscoring deep-rooted health-care inequalities. This necessitates urgent, targeted interventions to bridge this gap, focusing on improved access and trust building in health care for Black communities. Addressing these disparities is essential for equitable prostate cancer care in the postpandemic landscape.

## Supplementary Material

pkae016_Supplementary_Data

## Data Availability

The dataset used in this study is publicly available at https://www.cdc.gov/brfss.
